# Genetic polymorphisms linked to extreme postorthodontic external apical root resorption in Koreans

**DOI:** 10.1186/s40510-024-00521-7

**Published:** 2024-06-10

**Authors:** Jing Liu, Kwanwoo Park, Yoon Jeong Choi, Ji Hyun Lee, Jung-Yul Cha

**Affiliations:** 1 Department of Orthodontics, Institute of Craniofacial Deformity, Yonsei University College of Dentistry, Seoul, Korea; 2 Department of Biomedical and Pharmaceutical Sciences, Graduate School, Kyung Hee University, Seoul, Korea; 3 Department of Clinical Pharmacology and Therapeutics, College of Medicine, Kyung Hee University, 26, Kyungheedae-ro, Dongdaemun-gu, Seoul, 02447 Republic of Korea; 4 BK21 FOUR Project, Yonsei University College of Dentistry, Seoul, Korea; 5 Institute for Innovation in Digital Healthcare, Yonsei University, 50-1 Yonseiro, Seodaemun-gu, Seoul, 03722 Korea

**Keywords:** Genetic polymorphisms, External apical root resorption, Orthodontic treatment

## Abstract

**Background:**

External apical root resorption (EARR) is a common undesirable outcome of orthodontic treatment, this study aimed to identify genetic polymorphisms associated with the susceptibility to extreme orthodontic-induced EARR in a Korean population using extreme phenotype analysis sampling.

**Methods:**

Genomic DNA was isolated from the saliva of 77 patients who underwent orthodontic treatment involving two maxillary premolar extractions. The patients were divided into two groups based on EARR values measured on periapical radiographs: The significant resorption group (SG, EARR ≥ 4 mm) and the normal group (NG, EARR < 2 mm). In the NG group, patients with EARR < 1 mm were named the non-resorption group (NonG). Targeted next-generation sequencing was performed using the screened single nucleotide polymorphisms (SNPs), and firth logistic regression analysis was used to determine genetic associations with EARR. Haplotype-based association analysis was performed for specific SNPs.

**Results:**

SNPs related to genes *TNFSF11*, *TNFRSF11B*, *WNT3A*, *SFRP2, LRP6*, *P2RX7*, and *LRP1* were found to be significantly associated with severe EARR (*p* < 0.05, pre-Bonferroni correction *p*-values). Additionally, the haplotype CCA of rs17525809, rs208294, and rs1718119 *P2RX7* had a higher frequency in the SG group.

**Conclusion:**

Extreme phenotype analysis has identified eleven SNPs related to genes *TNFSF11*, *TNFRSF11B*, *WNT3A*, *SFRP2*, *LRP6*, *P2RX7*, and *LRP1* that are associated with severe root resorption in the Korean population. These findings will contribute to the development of predictive diagnostic tools for identifying severe root resorption that may occur during orthodontic treatment.

**Supplementary Information:**

The online version contains supplementary material available at 10.1186/s40510-024-00521-7.

## Background

**Fig. 1:**
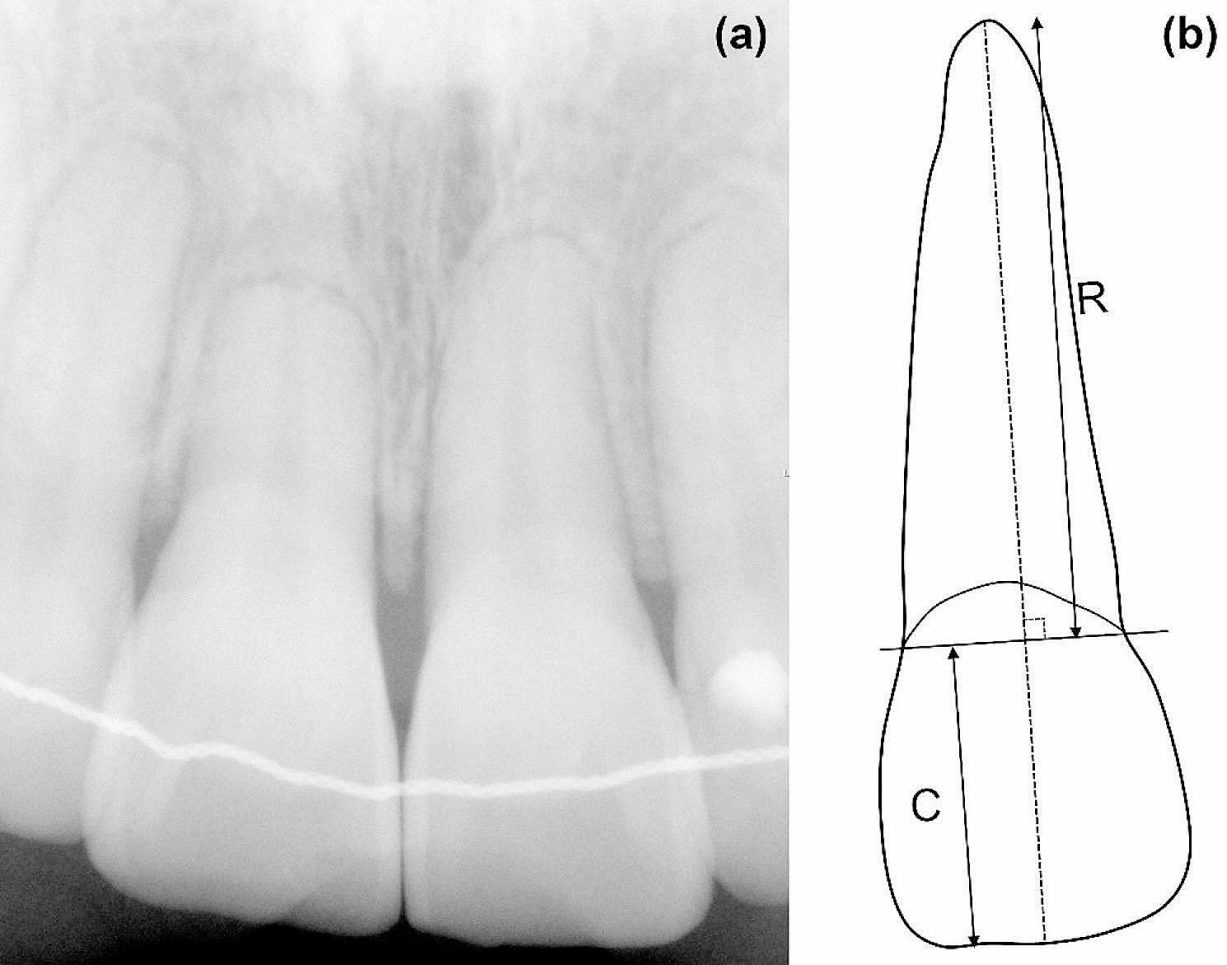
Example of external apical root resorption induced by orthodontic treatment on periapical radiograph and simple schematic diagram of root resorption measurement. (**a**) Periapical radiograph of a patient with short root after orthodontic treatment. (**b**) Simple schematic diagram showing lines related to the external apical root resorption measurements. The crown length (**C**) and root length (R) were measured as the longest distance from the incisal edge and apex to the line connecting the mesial and distal dentoenamel junction (DEJ) points

External apical root resorption (EARR) is a common adverse outcome of orthodontic treatment, most commonly affecting the upper incisors [1]. It is typically asymptomatic and appears shortened and blunted with rounded apices on radiographs [2] (Fig. 1a). A recent prospective study using cone-beam computed tomography (CBCT) showed that 94% of orthodontic patients had at least one root with an EARR greater than 1 mm, and 6.6% had over 4 mm [3].

Although a long-term evaluation study has shown that teeth that undergo EARR remain stable after active orthodontic treatment [4] and clinicians are advised to deliver a normal retention protocol to patients with EARR post-treatment [5], irreversible defects cannot regenerate to their original state.

Identifying the factors associated with EARR and mitigating its risks has been an ongoing topic of discussion over the years. Numerous factors have been detected in previous studies, including age at initial treatment, sex, root morphology, skeletal classification, history of trauma and endodontic treatment, duration of orthodontic active therapy, direction of tooth movement vector, extraction, use of rapid palatal expander, and host genotype [5–7]. Among these patient- and treatment-related factors, extraction treatment was found to result in a significantly greater incidence of EARR in the upper anterior teeth compared to non-extraction treatment, and treatment duration was found to be significantly linked to the amount of EARR in the meta-regression analysis [6–8].

Genetics is another patient-related factor that contributes to EARR. Genetic predisposition to EARR was first demonstrated by Harris et al. in 1997 using a sibling-pair model, but detailed information about the susceptibility genes was not listed [9]. Over the past two decades, the effects of various genetic polymorphisms on EARR have been studied in various populations. Most genes were associated with two pathways: (1) the ATP/P2RX7/IL-1B inflammation modulation pathway, and (2) the RANK/RANKL/OPG pathway [10]. Additionally, the Wnt signaling pathway, which mediates the OPG/RANKL ratio, may also be responsible for EARR [11]. A recent systematic review reported a low certainty level regarding the involvement of *P2RX7* (rs208294) in the risk of developing EARR [12]. Another gene, *IL-1α*, was found to be notably associated with EARR only in German Caucasians, despite being detected in four populations (German, Czech, Hispanics, and US Caucasians) [13–17]. Other single nucleotide polymorphisms (SNPs) have shown conflicting results in different ethnic groups [10].

These inconsistent results observed in various studies regarding EARR susceptibility may be attributable to population stratification, different target SNP panel sizes, or uncontrolled environmental variables. In addition, the method used to measure EARR, the grouping method based on the EARR amount, and the statistical analysis design can also influence the outcomes. To the best of our understanding, there is currently a lack of robust scientific evidence showing susceptible SNPs associated with EARR using extreme phenotype sampling designs, particularly within Asian populations. In order to achieve enhanced statistical power for detecting genetic effects in small sample sizes, we conducted extreme phenotype sampling (EPS) in this study [18].

Therefore, this study aimed to identify genetic polymorphisms associated with EARR susceptibility in a Korean population using targeted next-generation sequencing and extreme phenotype sampling designs.

## Materials and methods

### Participants

This study utilized the genetic information and clinical records collected as part of an orthodontic cohort study [19]. Of the 117 patients who underwent fixed orthodontic treatment at the Department of Orthodontics, OOO University, from February 2008 to May 2020, 77 individuals with no known biological relationships to each other were finally enrolled in this study. Briefly, the inclusion criteria were as follows: (1) orthodontic treatment with fixed appliances in the permanent dentition; (2) two maxillary premolars extracted and anterior teeth retracted for space closure; (3) periapical and lateral cephalometric radiographs of pre- and post-treatment are available; (4) no mini-screw-aided rapid palatal expansion (MARPE), orthognathic surgery, or periodontal surgery; and (5) no systemic diseases affecting tooth movement and bone metabolism.

This retrospective study was approved by the Institutional Review Board of the Yonsei University Dental Hospital (No. 2-2016-0023). Written consent was obtained from the patient or the patient’s parents before enrollment.

### EARR measurement and clinical characteristics collection

The EARR value was measured on periapical radiographs using a distance tool in a DICOM viewer (Zetta PACS, Taeyoung Soft Co. Ltd.), following the protocol proposed by Linge and Linge [20] and used in other published study [21]. In this method, a reference coordinate was established by initially creating a line that connects the mesial and distal dentoenamel junctions (DEJ) of the upper incisors. The greatest perpendicular distance from this line to the crown and the apical side was measured and recorded as the crown length (C) and root length (R). A schematic representation of this process is shown in Fig. 1b. The magnification factor difference of the radiographs between the two time points was adjusted by calculating a correction ratio of two crown lengths that remained relatively unchanged, and the absolute reduction of root length (mm), which was recorded as EARR in this study, was calculated using the formula below:

$$EARR={R}_{1}-\left({R}_{2}\times \frac{{C}_{1}}{{C}_{2}}\right)$$

R_1_, R_2_ represent the root length pre- and post-treatment, respectively

C_1_, C_2_ represent the crown length pre- and post-treatment, respectively

The EARR of the four upper incisors of each participant, except for incomplete tooth images of several lateral incisors, was measured, and the largest value was recorded as the EARR of that participant.

Other clinical characteristics of the participants were collected by measuring lateral cephalometric radiographs and screening electronic medical records. Parameters including age, sex, ANB, FMA, U1 to SN, crowding, displacement, overjet, overbite, and peer assessment rating score (PAR, unweighted and weighed) at the pre-treatment time point (T1) were collected, and leveling duration, retraction duration, leveling and retraction duration, total treatment duration, and anterior teeth retraction amount in the horizontal direction were counted and measured according to a previous study [19]. Displacement was defined as the sum of the absolute values of crowding or spacing to quantify the abnormal tooth positions. The retraction amount was determined as the difference in the anterior teeth edge in the horizontal direction during orthodontic treatment. This was assessed based on lateral cephalometric tracing using V-Ceph™ 5.5 (Osstem, Seoul, South Korea), in which we defined the horizontal reference plane (HRP) as the line 7° below the Sella–Nasion line [22].

### Measurement reliability

A reliability check for lateral tracing was not required because it was directly obtained from previously validated data. For EARR measurements, measurements from 23 randomly selected subjects (30%) were repeated after a 3-week interval, and the intra-examiner agreement of the proposed method was assessed using the intraclass correlation coefficient (ICC).

### Phenotyping

The subjects were classified according to the severity of EARR and divided into two groups. Patients with EARR equal to or greater than 4 mm after orthodontic treatment were classified into the significant resorption group (SG, *n* = 19), and patients who had less than 2 mm were classified into the normal group (NG, *n* = 36) [3, 14]. Furthermore, a cutoff of 1 mm EARR was used, and subjects who had EARR less than 1 mm were assigned to the non-resorption group (NonG, *n* = 16) [1]. This group was subjected to subgroup analysis by comparing it to the significant resorption group.

### Genetic analysis

Genomic DNA was isolated from collected saliva and targeted next-generation sequencing was performed using screened single nucleotide polymorphisms (SNPs) [23]. Specifically, previously reported EARR-related SNPs and Wnt signaling pathway-related genes were selected (Supplementary Table 1). For targeted next-generation sequencing analysis, only the coding DNA sequence (CDS) region of the genes was included; for SNPs other than those in the CDS region, additional target probes were designed. Hybridization capture-based next-generation sequencing was performed. Next-generation sequencing data were obtained using a genome analysis tool kit. Sequencing reads obtained from Illumina NextSeq 500 platforms were further analyzed using BWA-MEM, Picard (v1.115), Samtools (v1.1), and GATK (v4.0.4.0) was used to call single nucleotide variants. Targeted capture sequencing identified 299 variants in the candidate regions involved in EARR. 6 of those variants, with an allele frequency of 0 or 100%, were excluded due to their monomorphic nature, and the remaining 293 variants were analyzed.

### Statistical analysis

Statistical analyses were performed using R 4.2.2 (R Foundation) and GraphPad Prism 9.5.0 software. Differences in clinical parameters between SG and NG and between SG and NonG were analyzed using the χ2 test or t-test, as appropriate. Firth logistic regression analysis was performed to determine the genetic association with EARR using the allele model. All *P*-values were based on two-sided comparisons, and *P* < 0.05 was considered statistically significant.

### Bioinformatic analysis

Linkage disequilibrium (LD) analysis was performed on the SNPs within each gene, identifying significant LD among the SNPs in *TNFSF11* and *P2RX7*. Consequently, LD plots for these SNPs were generated using Haploview 4.2 software to assist in tagging SNP selection (Supplementary Fig. 1). Haplotype-based association analysis of *P2RX7* 3 SNPs was performed using Plink (version 1.07, http://pngu.mgh.harvard.edu/purcell/plink/) [24]. The protein-protein interaction networks were performed using STRING 11.5 [25], and the K-means clustering algorithm was used to identify the groups.

## Results

### Measurement reliability

The proposed method for EARR assessment on periapical radiographs demonstrated high reliability, with an ICC of 0.92 (95% CI:0.82–0.96).

### Demographic and clinical characteristics

Table 1 (NG and SG) and Supplementary Table 2 (NonG and SG) present the demographic and clinical characteristics of the enrolled subjects categorized by phenotype. Most of the participants were female (83.3% in NG and 78.9% in SG) with an average age of 20.15 years in the NG group and 19.7 years in the SG group. The initial characteristics and improvements brought about by orthodontic treatment were relatively homogeneous across the groups, except for the amount of EARR. The SG group had an average EARR of 5.3 ± 1.5 mm during treatment, while the NG group had an EARR of 1.1 ± 0.6 mm. In addition, the NonG group showed a gain of 0.6 ± 0.3 mm EARR at the debonding time point.

**Table 1:** The clinical characteristics of the enrolled subjects

Clinical parameters	Normal group (*n* = 36)	Significant resorption group (*n* = 19)	*p*-value* (Normal group vs. Significant resorption group)
Age (years), median (range)	20.15 (12-46.9)	19.7 (12-36.5)	0.94
Gender, *N* (%)			
Female	30 (83.3)	15 (78.9)	0.72
Male	6 (16.7)	4 (21.1)	
Leveling Duration (month), mean ± SD	9.4 ± 5.2	10.5 ± 6.3	0.53
Retraction Duration (month), mean ± SD	18.3 ± 7	17 ± 8.3	0.40
Leveling Duratio + Retraction Duration (month), mean ± SD	27.7 ± 6.2	27.5 ± 6.8	0.64
Total duration (month), mean ± SD	35 ± 7.9	33.8 ± 8.4	0.53
Horizontal anterior retraction	4.4 ± 2.9	5.1 ± 2.5	0.35
EARR, mean ± SD	1.1 ± 0.6	5.3 ± 1.5	**9.06E-11**
ANB(T1)	3.8 ± 2.2	4.8 ± 2.7	0.09
FMA(T1)	29.3 ± 6.1	29.5 ± 5.8	0.93
U1 to SN(T1)	106.9 ± 8.5	107 ± 6.2	0.93
Crowding (mm)	3 ± 2.7	4.4 ± 4.1	0.19
Displacement (mm)	3.3 ± 2.4	4.4 ± 4.1	0.27
Overjet (mm)	3.6 ± 2.1	2.9 ± 2.4	0.39
Overbite (mm)	1.7 ± 2.2	1.7 ± 2.4	0.94
PAR (unweighted)	5.2 ± 2.6	5.6 ± 2.8	0.61
PAR (weighed)	16.5 ± 9.7	16.3 ± 9.4	0.87

* The χ^2^ test or t-test was used appropriately. Bold values denote statistical significance at *p* < 0.05

### Allelic association

In the analysis of extreme phenotypes within the SG and NG groups, we employed Firth’s logistic regression to examine 293 variants. Following Bonferroni correction for multiple testing (with a cut-off *P* value of 0.05/293 = 1.71 × 10^–4^), none of these variants showed a significant association with the extreme EARR phenotype. However, seven independent loci did achieve significance (*p* < 0.05, pre-Bonferroni correction *p*-values) in our analysis of these groups’ extreme phenotypes. Table 2 lists theirallele frequencies. Specifically, the C allele of the *P2RX7* rs17525809 polymorphism was not carried by any participant in the NG group, whereas it was found more frequently (10.5%) in the SG group.

**Table 2:** SNPs significantly associated with EARR in extreme phenotype analysis

Gene	RSID of SNP	Base change* (AA change)	allele	Normal group (*N* = 36)	Significant resorption group (*N* = 19)	*p*-value
*TNFSF11*	rs2296533 ^a^	c.126T > C (p.Pro42Pro)	T	47 (69.1)	17 (44.7)	**0.009**
			C	21 (30.9)	21 (55.3)	
*WNT3A*	rs4653533	c.580–2894 C > T	C	63 (87.5)	23 (60.5)	**0.001**
			T	9 (12.5)	15 (39.5)	
*SFRP2*	rs3810765	c.502 + 6 C > T	C	35 (48.6)	27 (71.1)	**0.022**
			T	37 (51.4)	11 (28.9)	
*P2RX7*	rs17525809	c.227T > C (p.Val76Ala)	T	72 (100.0)	34 (89.5)	**0.012**
			C	0 (0.0)	4 (10.5)	
	rs208294	c.463T > C (p.Tyr155His)	T	48 (66.7)	16 (42.1)	**0.015**
			C	24 (33.3)	22 (57.9)	
	rs1718119 ^b^	c.1042G > A (p.Ala348Thr)	G	70 (97.2)	31 (81.6)	**0.008**
			A	2 (2.8)	7 (18.4)	
*LRP1*	rs1800137	c.1209 C > T (p.Thr403Thr)	C	52 (72.2)	34 (89.5)	**0.045**
			T	20 (27.8)	4 (10.5)	

*p*-values were pre-Bonferroni correction *p*-values obtained from firth’s logistic regression analysis (with sex, age, and total duration as covariates). Bold values denote statistical significance at *p* < 0.05. *Nucleotide location numbers were assigned according to *TNFSF11* (NM_003701.3), *WNT3A* (XM_005273340.1), *SFRP2* (NM_003013.2), *P2RX7* (NM_002562.5), and *LRP1* (NM_002332.2) mRNA sequences. The a and b denote tagged SNPs

In the subgroup analysis of the SG and NonG groups, 10 independent loci with significant associations (*p* < 0.05, pre-Bonferroni correction *p*-values) were detected. Table 3 lists their allele frequencies. Compared to the results of the extreme phenotype analysis (Table 2), four additional SNPs were found (rs3742257 located in *TNFSF11*, rs2875845 located in *TNFRSF11B*, rs2302685 located in LRP6, and rs1800141 located in *LRP1*). In total, seven genes were identified in these two analyses.

**Table 3:** Subgroup analysis on the association of EARR between the Non-resorption group and Significant resorption group

Gene	RSID of SNP	Base change* (AA change)	Allele	Non-resorption group (*n* = 16)	Significant resorption group (*n* = 19)	*p*-value
*TNFSF11*	rs2296533	c.126T > C (p.Pro42Pro)	T	22 (78.6)	17 (44.7)	**0.009**
			C	6 (21.4)	21 (55.3)	
	rs3742257	c.388-1690T > C	T	10 (31.2)	21 (55.3)	**0.044**
			C	22 (68.8)	17 (44.7)	
*TNFRSF11B*	rs2875845	c.31-4229T > C	T	26 (81.3)	36 (94.7)	**0.037**
			C	6 (18.7)	2 (5.3)	
*WNT3A*	rs4653533	c.580–2894 C > T	C	28 (87.5)	23 (60.5)	**0.014**
			T	4 (12.5)	15 (39.5)	
*LRP6*	rs2302685	c.3184G > A (p.Val1062Ile)	G	7 (21.9)	2 (5.3)	**0.032**
			A	25 (78.1)	36 (94.7)	
*P2RX7*	rs17525809	c.227T > C (p.Val76Ala)	T	32 (100.0)	34 (89.5)	**0.040**
			C	0 (0.0)	4 (10.5)	
	rs208294	c.463T > C (p.Tyr155His)	T	22 (68.8)	16 (42.1)	**0.011**
			C	10 (31.2)	22 (57.9)	
	rs1718119 ^a^	c.1042G > A (p.Ala348Thr)	G	32 (100.0)	31 (81.6)	**0.003**
			A	0 (0.0)	7 (18.4)	
*LRP1*	rs1800137	c.1209 C > T (p.Thr403Thr)	C	21 (65.6)	34 (89.5)	**0.024**
			T	11 (34.4)	4 (10.5)	
	rs1800141	c.8142G > A (p.Thr2714Thr)	G	22 (68.8)	35 (92.1)	**0.012**
			A	10 (31.3)	3 (7.9)	

*p*-values were pre-Bonferroni correction *p*-values obtained from firth’s logistic regression analysis (with sex, age, and total duration as covariates). Bold values denote statistical significance at *p* < 0.05. *Nucleotide location numbers were assigned according to *TNFSF11* (NM_003701.3), *TNFRSF11B* (NM_002546.3), *WNT3A* (XM_005273340.1), *LRP6* (NM_002336.2), *P2RX7* (NM_002562.5), and *LRP1* (NM_002332.2) mRNA sequences. The a denotes tagging SNPs.

The haplotype association analysis was performed to detect the association between the three SNPs located in *P2RX7* and significant EARR. Haplotype CCA, comprising *P2RX7* c.227T/C (rs17525809, Val76Ala), c.463T/C (rs208294, Tyr155His), and c.1042G/A (rs1718119, Ala348Thr), was significantly more frequent in SG than in NG, whereas haplotype TTG, comprising these three, was less frequent in the SG group than in the NG group (Table 4). Supplementary Table 3 shows consistent results when comparing the SG and NonG groups.

**Table 4:** Haplotype association analysis between *P2RX7* SNP haplotypes and significant resorption versus normal groups

Haplotype			Frequency		*p*-value
rs17525809	rs208294	rs1718119	Significant resorption group	Normal group	
C	C	A	0.1053	0	**0.00504**
T	C	A	0.07895	0.02778	0.2205
T	C	G	0.3947	0.3056	0.3465
T	T	G	0.4211	0.6667	**0.01302**

Haplotype-based association analyses were performed using PLINK software. Underlined text indicates a variant of each SNP. Bold values denote statistical significance at *p* < 0.05

### K-means clustering

**Fig. 2:**
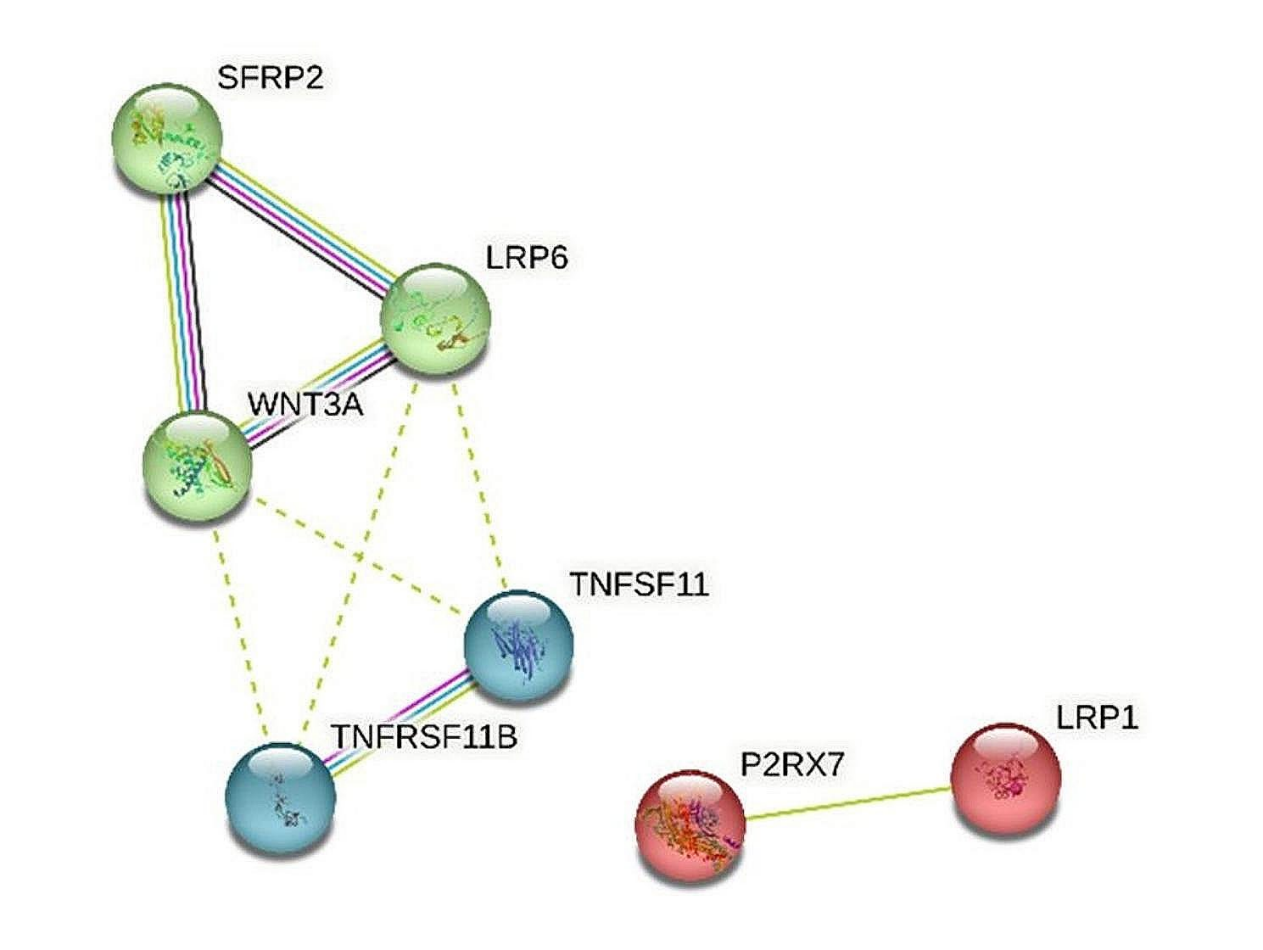
Outcomes of string analysis using the K-means clustering algorithm. String analysis using the K-means clustering algorithm shows that significantly associated genes are involved in known and predicted protein-protein interactions. The network nodes stand for those genes shown in Tables 2 and 3. Different colored lines represent six types of evidence used to predict associations. Green line: neighborhood evidence; blue line: co-occurrence evidence; purple line: experimental evidence; yellow line: text mining evidence; light blue line: database evidence and black line: coexpression evidence

String analysis using the K-means clustering algorithm showed that significantly associated genes were involved in known and predicted protein-protein interactions. Figure 2 depicts three clusters, with different colored lines representing the six types of evidence used to predict associations, one of which comprised genes *SFRP2, WNT3A*, and *LRP6*, whereas the second comprised genes *TNFSF11* and *TNFRSF11B*. The third cluster comprised two genes, *P2RX7* and *LRP*.

## Discussion

In this study, we collected and analyzed the clinical data and biological DNA information from 77 patients who received orthodontic treatment involving premolars extraction. This research delved into the correlation between host genetic variants and significant EARR using an extreme phenotype sampling design. We conducted two sets of comparisons among the three groups to estimate the differences in clinical variables and EARR was the sole variable that displayed significant differences in both comparisons. The SG group had slightly higher values for horizontal anterior retraction amount, ANB at T1, and crowding at T1 than the NG and NonG (*p* > 0.05).

The radiographs used in this study were periapical images that are easy to obtain, have quick access, and are routinely used for orthodontic treatment. Previous research has shown that cone-beam computed tomography (CBCT) is reliable in detecting the extent of EARR, whereas periapical radiography may underestimate the value. Nevertheless, no significant difference was found in the detection of severe EARR between the measurements acquired using the two radiographic methods, and exercise caution is advised when opting for CBCT if other radiographs are available [26]. Moreover, periapical films are recommended over panoramic films, as the latter may overestimate root loss by up to 20% or more [27]. To achieve accurate measurements of root resorption, the rule-of-three formula proposed by Linge and Linge [20] was used to correct for angular changes during filming [28].

After collecting saliva samples, targeted next-generation sequencing was performed using a panel of 35 target genes and 58 previously reported loci outside the exonic regions. Most previous studies evaluated a limited number of genetic polymorphisms and assessed whether these SNPs are associated with specific populations. A genome-wide association study (GWAS) was conducted with over 10,000 SNPs within chromosomes 2, 4, 8, 12, 18, X, and Y and identified 27 novel genetic variants with marginal association values, specifically located in the sexual chromosomes [29]. Besides previously reported SNPs, this study explored genes related to the Wnt signaling pathway. This pathway is known to be essential for bone development and homeostasis [30], periodontal ligament [31], is suspected to be involved in periodontitis [32, 33], and is especially involved in the secretion of new dentin after tooth injury [34].

Three genes related to Wnt signaling exhibited nominal association with EARR (Tables 2 and 3, and Fig. 2). One of these genes, *SFRP2* (Secreted Frizzled Related Protein 2), is a member of the *SFRP* family of proteins that inhibits the Wnt signaling pathway and participates in tooth formation. Previous research has indicated that *SFRP2* promotes osteo/odontogenic differentiation [35] and plays an important role in osteoblast differentiation [36]. Moreover, it offers a promising cytokine candidate for enhancing tissue regeneration in hypoxic and inflammatory niche [35]. However, the biological mechanisms of how *SFRP2* rs3810765 affect osteoblast differentiation in localized hypoxia caused by compression orthodontic force remains unclear. Another gene associated with Wnt signaling, the Wnt ligand *WNT3A*, is reported to impede cementoblast differentiation and promote cell proliferation and has been proven to be related to periodontal tissue remodeling during orthodontic tooth movement [37, 38]. Root resorption induced by orthodontic force more frequently and severely damages apical cellular cementum. It’s noteworthy that canonical Wnt/β-catenin signaling positively influences osteogenesis but may adversely affect cementogenesis [39]. *WNT3A* rs4653533 was previously associated with higher bone mineral density (BMD) in total hip [40] and in this study, the T allele of rs4653533 was significantly associated with EARR. The Low-density lipoprotein receptor-related protein 6 (*LRP6*) gene was recently revealed to be linked with autosomal dominant inherited tooth agenesis [41], and a mutation at rs2302685 was previously associated with Alzheimer’s disease [42], LDL-cholesterol [43], and bone mass [44]. This mutation results in an amino acid substitution (c.3184G > A, p.Val1062Ile), and previous research has revealed that *LRP6*Val-1062 exhibits decreased activity of β-catenin signaling, which leads to abnormal differentiation from mesenchymal progenitors to osteoblasts [42].

*TNFSF11* and *TNFRSF11B* encode RANKL and OPG, respectively. The OPG/RANKL/RNAK pathway is critical for root resorption during orthodontic tooth movement, as it regulates osteoclast differentiation and maturation [45, 46]. In a mouse study, early and severe root resorption was observed in OPG-KO mice by enhancing osteoclast activation and decreasing the mineralization of cementum [47]. OPG acts as a decoy receptor, inhibiting the RANKL/RANK interaction and thus preventing osteoclast maturation, which in turn protects bone mass [48]. In the mouse study, overly activated osteoclasts were found on the surface of cementum in OPG-KO mice and osteoclastic markers protein expression increased significantly at the molecular level. The result suggested OPG might protect cementum against resorption through the same mechanism as in bone resorption. This study found an increase in the frequency of allele C of *TNFSF11* rs2296533 and a decrease in the frequency of allele C of *TNFSF11* rs3742257 in the SG; however, related research on these SNPs is insufficient. Polymorphisms in *TNFSF11* were detected in a study of white patients conducted by Castilhos et al., and rs12455775 was found to be significantly associated with EARR [49]. This difference in polymorphisms could be explained by racial diversity. *TNFRSF11B* rs2875845 was found to be nominal associated with EARR in this study, which is consistent with the results reported by Castilhos et al. However, the frequencies of T and C alleles showed opposite tendencies. This SNP is located in a deep intron and, although it does not encode a protein, is predicted to create or break binding sites for transcription factors [50]. *P2RX7* encodes a purinergic receptor that is activated by adenosine triphosphate (ATP) and is involved in bone and root remodeling by regulating hyalinized tissue metabolism via the ATP-P2RX7-IL1 pathway [45, 51]. *P2x7r* KO-derived macrophages do not release IL-1 in response to ATP, leading to a diminished acute inflammatory response that ultimately results in abundant apoptotic and necrotic cells, and generalized tissue damage [52], it was proven in mice study that the lack of *P2x7r* caused about a 20% increase in root resorption in the KO mice [51]. This study found that three SNPs in relation to *P2RX7* were associated with severe EARR and resulted in amino acid sequence changes at positions 76, 155, and 348. The polymorphism rs208294 was recently shown to be associated with EARR in a systematic review; however, the level of evidence presented is limited [12].

According to the haplotype association analysis shown in Table 4, the haplotype CCA of rs17525809, rs208294, and rs1718119 had a higher frequency in SG than in NG, whereas the TTG type had a lower frequency (*p* < 0.05). Although this outcome was not statistically significant when comparing the SG and NonG groups, the trend was consistent. This may be attributed to the comparatively smaller sample size of the NonG in contrast to the NG. Among all SNPs identified in the human *P2RX7* gene, some are known to act as a gain-of-function polymorphism, with the most recurrent one being the 463 C > T (rs208294, His155 into Tyr), which increases IL-1β and IL-18 secretion [53]. When combined with another variant, rs1718119 (Ala348Thr), the 348Thr variant, as with the 155Tyr variant, markedly elevated the maximum responses for pore and channel functions, and drove more P2X7 protein to be expressed [54].

In contrast, the 76Ala variant combined with 155His leads to the least permeability for calcium entry compared to other haplotypes, which represents a loss of function [55]. Other *P2RX7*-related studies in Autoimmune Encephalomyelitis have shown that blocking ATP P2X7 receptors prevents ATP excitotoxicity [56]. In the present study, the combination of 76Ala, 155His, and 348Thr may lead to a loss of function and result in more severe root resorption. This result is consistent with that of a knockout mouse study that showed that the absence of *P2RX7* gene increased external root resorption during orthodontic treatment [51].

The strength of this study lies in its examination of the effects of genetic variations in the host through extreme phenotype analysis in the Korean population and its novelty in assessing the relevant *P2RX7* haplotype associated with EARR. However, further studies involving CBCT for more accurate resorption measurements and larger sample sizes are necessary to confirm the associated SNPs. These results offer new insights for clinicians to enhance their understanding of the factors contributing to root resorption. Although the associations identified did not retain statistical significance after correction for multiple testing (*p* > 0.05), suggesting that the findings should be interpreted with caution, these data highlight the complexity of root resorption as a trait influenced by various genetic factors. This also indicates the necessity of further investigation into these variations we studied in larger populations to achieve the statistical power required for more definitive conclusions. Additionally, these findings provide genetic insights that could be considered in the future development of predictive models concerning EARR. On the other hand, the identification of these associated genes enables researchers to conduct experiments at animal or cellular levels to uncover deeper biological mechanisms, ultimately assisting in screening risk factors in clinical work. Nonetheless, it should be noted that EARR has a multifactorial effect that requires continuous monitoring, and patients without associated risk variants may experience it.

## Conclusions

In conclusion, this study used an extreme phenotype sampling design to assess genetic variants associated with susceptibility to severe external apical root resorption induced by orthodontic treatment. The results revealed that SNPs related to gene *TNFSF11, TNFRSF11B, WNT3A, SFRP2, LRP6, P2RX7*, and *LRP1* were associated with severe EARR, and for the first time, identified that the haplotype CCA of rs17525809, rs208294, rs1718119 had a higher frequency in significant resorption group. However, it is imperative to conduct these analyses using larger sample sizes to ensure the reliability of the findings and further functional analyses are required to confirm the involvement of these novel genes.

### Electronic supplementary material

Below is the link to the electronic supplementary material.

Supplementary Material 1

## Data Availability

The accession number for the SRA data is PRJNA951213.

## Abbreviations

TNFSF11: TNF Superfamily Member 11; also known as Receptor Activator Of Nuclear Factor Kappa B Ligand, RANKL
TNFRSF11B: TNF Receptor Superfamily Member 11b; also known as Osteoprotegrin, OPG
WNT3A: Wnt Family Member 3A
SFRP2: Secreted Frizzled Related Protein 2
LRP6: Lipoprotein Receptor-Related Protein 6
P2RX7: Purinergic Receptor P2X, Ligand-Gated Ion Channel, 7; also abbreviated as *P2 X7*
LRP1: Lipoprotein Receptor-Related Protein 1
IL-1α: Interleukin-1 Alpha
