# Supplementary material for: Genetic polymorphisms linked to extreme postorthodontic external apical root resorption in Koreans
Source: Prog Orthod. 2024 Jun 10;25:23. doi: 10.1186/s40510-024-00521-7 (PMC11162991; doi:10.1186/s40510-024-00521-7)
Supplement: Supplementary file 1 — Supplementary Material 1 [file 40510_2024_521_MOESM1_ESM.docx]

**Supplementary Figure 1**

**
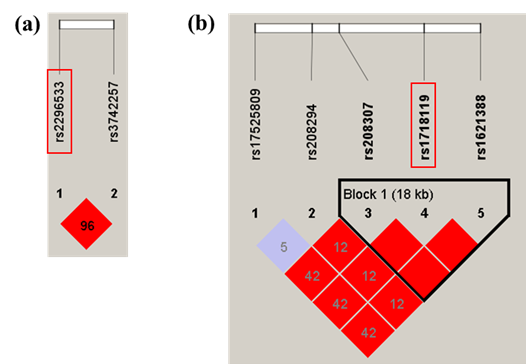
**

**Supplementary Figure 1**. Linkage disequilibrium plot of (a) TNFSF11 and (b) P2RX7 SNPs generated by Haploview software. Linkage disequilibrium (LD) is displayed as pairwise r2 values. The shading represents the magnitude and significance of pairwise LD, with a red-to-white gradient reflecting higher-to-lower LD values. The red diamond without a number corresponds to r^2^ values of 1.0. The tagging SNPs selected in each block are shown by red box.

**Supplementary Table 1.** List of targeted sequencing regions

| ***Target genes*** | *ALPL, CASP1, CASP5, DKK1, DKK2, DKK3, FRZB, FZD7, IL-17A, IL1A, IL1B, IL1RN, IL-6, IL-8, IRAK1, LRP1, LRP5, LRP6, P2RX7, SFRP1, SFRP2, SFRP4, SFRP5, SOST, SPP1, TNF, TNFRSF11A, TNFRSF11B, TNFSF11, VDR, WIF1, WISP3, WNT10B, WNT3A, WNT7B* |
| --- | --- |
| ***Previously reported known loci in outside the exonic regions*** | rs1038434, rs12585229, rs3742257, rs931273, rs11730582, rs9138, rs1230399, rs851054, rs851056, rs12455775, rs12956925, rs12959396, rs12970081, rs17069845, rs17069898, rs17069902, rs17069904, rs17720953, rs3826620, rs4426449, rs4485469, rs4500848, rs4524034, rs4941125, rs4941129, rs6567272, rs7233197, rs7236060, rs7237982, rs7239667, rs8083511, rs8086340, rs8089829, rs8099222, rs9951012, rs16890444, rs1800587, rs1800629, rs1800796, rs2275913, rs3102735, rs1032128, rs11573856, rs11573884, rs11573901, rs11573938, rs1485289, rs2875845, rs3102724, rs3102728, rs3134057, rs3134060, rs7010267, rs3242, rs4653533, rs752107, rs530537, rs554344 |

| **Supplementary Table2.** The clinical characteristics of subgroup patients | | | |
| --- | --- | --- | --- |
| **Clinical parameters** | **Non-resorption group (n=16)** | **Significant resorption group (n=19)** | ***p*-value* (Non-resorption group vs Significant resorption group)** |
| Age (years), median (range) | 20.45 (12-41.6) | 19.7 (12-36.5) | 0.89 |
| Gender, N (%) |  |  |  |
| Female | 14 (87.5) | 15 (78.9) | 0.67 |
| Male | 2 (12.5) | 4 (21.1) |  |
| Leveling Duration(month), mean±SD | 9.6±5.8 | 10.5±6.3 | 0.68 |
| Retraction Duration(month), mean±SD | 15.4±6.7 | 17±8.3 | 0.64 |
| Leveling Duratio+Retraction Duration(month), mean±SD | 25±5.6 | 27.5±6.8 | 0.49 |
| Total duration(month), mean±SD | 30.8±6.1 | 33.8±8.4 | 0.22 |
| Horizontal anterior retraction | 4±2.8 | 5.1±2.5 | 0.22 |
| EARR, mean±SD | 0.6±0.3 | 5.3±1.5 | **1.89E-11** |
| ANB(T1) | 3.8±1.9 | 4.8±2.7 | 0.14 |
| FMA(T1) | 28.4±5.3 | 29.5±5.8 | 0.77 |
| U1 to SN(T1) | 107.1±9.3 | 107±6.2 | 0.97 |
| Crowding(mm) | 3.7±2.7 | 4.4±4.1 | 0.54 |
| Displacement(mm) | 3.7±2.7 | 4.4±4.1 | 0.54 |
| Overjet(mm) | 3.5±1.8 | 2.9±2.4 | 0.55 |
| Overbite(mm) | 1.1±2 | 1.7±2.4 | 0.54 |
| PAR(unweighted) | 5.7±2.4 | 5.6±2.8 | 0.97 |
| PAR(weighed) | 18.5±7.5 | 16.3±9.4 | 0.44 |

*The x2 test or t-test was used appropriately. The bold values denote statistical significance at *p* < 0.05.

**Supplementary Table3.** Haplotype association analysis between *P2RX7* SNP haplotypes and Significant resorption versus Non-resorption groups

| **Haplotype** | | |  | **Frequency** | | ***p*-value** |
| --- | --- | --- | --- | --- | --- | --- |
| **rs17525809** | **rs208294** | **rs1718119** |  | **Significant resorption group** | **Non-resorption group** |  |
| C | C | A |  | 0.1053 | 0 | 0.05874 |
| T | C | A |  | 0.07895 | 0 | 0.1042 |
| T | C | G |  | 0.3947 | 0.3125 | 0.4744 |
| T | T | G |  | 0.4211 | 0.6875 | **0.0258** |

Haplotype-based association analyses were performed using PLINK software. Underlined text indicates a variant of each SNP. Bold values denote statistical significance at *p* < 0.05.
